# The Role of Human Milk Lipids and Lipid Metabolites in Protecting the Infant against Non-Communicable Disease

**DOI:** 10.3390/ijms23147490

**Published:** 2022-07-06

**Authors:** Alexandra D. George, Satvika Burugupalli, Sudip Paul, Toby Mansell, David Burgner, Peter J. Meikle

**Affiliations:** 1 Metabolomics Laboratory, Baker Heart and Diabetes Institute, Melbourne 3004, Australia; satvika.burugupalli@baker.edu.au (S.B.); sudip.paul@baker.edu.au (S.P.); peter.meikle@baker.edu.au (P.J.M.); 2 Baker Department of Cardiometabolic Health, University of Melbourne, Parkville 3010, Australia; 3 Department of Cardiovascular Research, Translation and Implementation, La Trobe University, Bundoora 3086, Australia; 4 Murdoch Children’s Research Institute, Royal Children’s Hospital, Parkville 3052, Australia; toby.mansell@mcri.edu.au (T.M.); david.burgner@mcri.edu.au (D.B.); 5 Department of Paediatrics, University of Melbourne, Parkville 3010, Australia; 6 Department of Paediatrics, Monash University, Clayton 3800, Australia

**Keywords:** lipidomics, human milk, developmental origins of health and disease, infant programming

## Abstract

Non-communicable diseases continue to increase globally and have their origins early in life. Early life obesity tracks from childhood to adulthood, is associated with obesity, inflammation, and metabolic dysfunction, and predicts non-communicable disease risk in later life. There is mounting evidence that these factors are more prevalent in infants who are formula-fed compared to those who are breastfed. Human milk provides the infant with a complex formulation of lipids, many of which are not present in infant formula, or are present in markedly different concentrations, and the plasma lipidome of breastfed infants differs significantly from that of formula-fed infants. With this knowledge, and the knowledge that lipids have critical implications in human health, the lipid composition of human milk is a promising approach to understanding how breastfeeding protects against obesity, inflammation, and subsequent cardiovascular disease risk. Here we review bioactive human milk lipids and lipid metabolites that may play a protective role against obesity and inflammation in later life. We identify key knowledge gaps and highlight priorities for future research.

## 1. Introduction

The incidence of non-communicable diseases (NCD), including type 2 diabetes, cardiovascular disease and chronic obstructive pulmonary disease, is rapidly increasing and NCD are predicted to contribute to over 75% of deaths worldwide by 2030 [1]. The pathogenesis of most NCD begins in childhood, as early as the first 1000 days of life, and during this period of developmental and physiological plasticity, nutrition is a key determinant of long-term health outcomes [2]. Both primordial and primary prevention are essential to reduce the global burden of obesity and downstream NCD.

Key risk factors for NCD in adulthood are obesity and metabolic dysfunction, and adverse childhood adiposity is a risk factor for both obesity and metabolic dysfunction in adulthood [3]. There is considerable evidence that breastfeeding is protective against excess adiposity, with breastfed infants having lower adiposity and a 13–26% reduction in overweight and obesity compared to those who are not breastfed [4,5]. Breastfeeding occurrence and duration are inversely associated with both the extent and velocity of infant weight gain, and infants who are formula-fed have different growth trajectories and higher rates of obesity and diabetes later in life compared to their human milk-fed counterparts [6]. In addition, inflammation is increased in obesity and is a shared pathogenic mechanism for most NCD in adults [7]. Numerous factors contribute to inflammation, including rapid weight gain and infections, both of which are more common in formula-fed infants than those who are breastfed [4,8]. Reduction or modification of adiposity and inflammatory trajectories early in life, through breastfeeding, are of considerable interest and may help reduce NCD risk later in life. 

**Figure 1:**
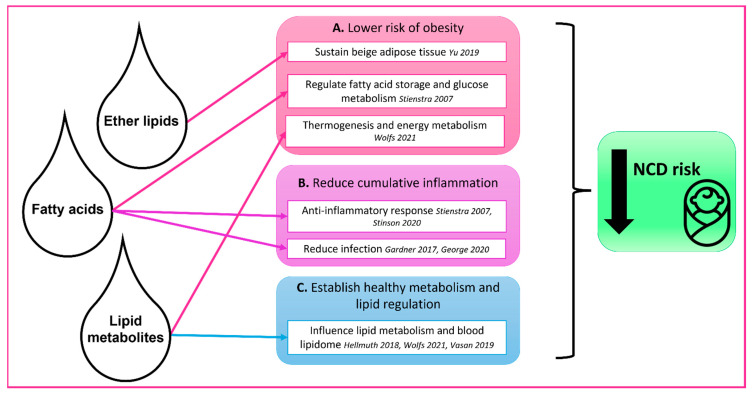
Summary of the possible roles of bioactive human milk lipids and lipid metabolites in protecting the infant against non-communicable disease risk. Lipids and lipid metabolites are delivered to the infant early in life through human milk and contribute to (**A**) lower risk of obesity, (**B**) reduction in cumulative inflammation, and (**C**) establishment of healthy metabolism and lipid regulation. Arrows indicate identified associations between human milk components and infant protection, as per the literature. Key articles included in the review are referenced [7,13,15,16,17,18,19,20].

The protective effects of breastfeeding on early and later disease risks may be driven, at least in part, by the unique and complex lipidome (lipid composition) of human milk. There is increasing evidence of the importance of lipids in human health and given the differences between human milk and infant formula lipid composition, as well as differences in the blood lipidome of breastfed and formula-fed infants, the lack of exposure to the human milk lipidome may contribute to metabolic dysfunction and the higher risk of NCD observed in formula-fed infants. As such, human milk lipidomic analyses are a promising approach to investigate protection against obesity, inflammation and later NCD [9,10,11,12]. There are several mechanisms through which bioactive lipid species may protect the newborn infant against NCD risk. For example, ether lipids such as alkyldiacylglcyerols may sustain thermogenic beige adipose tissue and thus reduce obesity, monoacylglycerides released from triacylglycerides may protect against infection and thus cumulative inflammation, while certain fatty acids may contribute to optimal infant lipid metabolism [13,14,15]. Such mechanisms are intimately related to infant growth and prime health for life (Figure 1). 

It should be noted that human milk contains a myriad of components that are involved in infant growth, health and development, and that lipids and lipid metabolites are only one component of interest. The biological relevance of the human milk lipidome, however, is relatively novel and under-investigated. Therefore, in this review we present the current knowledge on human milk lipids and lipid metabolites that may protect the breastfed infant against obesity, inflammation, and later life NCD. A literature search was made using variations of the following search terms (human milk, breastmilk, lipid, fat, outcomes, obesity, inflammation, metabolism) and research articles were selected if they included both measurement of lipids and/or lipid metabolites in human milk, and presentation of findings with regards to infant outcomes (key articles and associations are summarised in Figure 1) [7,13,16,17,18,19,20]. Due to the nature of this topic, very few articles met the search criteria; therefore, articles were supplemented as necessary throughout this review to discuss possible mechanisms and highlight future research priorities and considerations to identify and validate new findings.

## 2. Breastfeeding and Non-Communicable Disease Risk

There is an ever-growing body of evidence linking breastfeeding to a reduction in obesity risk [5,21,22,23]. The World Health Organization recommends breastfeeding the infant exclusively for the first six months of life and continued breastfeeding for up to two years post-partum [4,24]. Specifically, original research, meta-analyses and systematic reviews have identified that breastfeeding influences early life growth, inflammation, and metabolism. 

### 2.1. Early Life Growth

Early life is a critical period where adipose tissue increases and distributes throughout the body and risk of chronic disease is established [25]. Adipose tissue is composed of adipocytes, immune cells and other cell types, and brown, beige and white adipose tissue are present in infants. During early development, brown and beige adipose tissue predominate, while white adipose tissue predominates in adulthood and has the key function of energy storage rather than thermogenesis [2]. This change from predominantly brown and beige adipose tissue to predominantly white adipose tissue is accelerated in obese children, and premature loss of beige adipose tissue has been proposed as a potential cause of obesity (Figure 1A) [13]. Adiposity and growth trajectories differ between infants who are exclusively breastfed, mixed-fed, or exclusively formula-fed. Children who are breastfed have more beige adipose tissue, and increased weight, length and fat deposition in the first few months of life, then slowed growth to one year of age. In contrast, formula-fed infants have less beige adipose tissue and more rapid weight gain throughout the first year of life [26]. Recent findings indicate an inverse relationship of breastfeeding duration with infant weight gain and body-mass index (BMI), and that rapid growth in the first year is associated with a high fat mass and central fat distribution, and therefore obesity risk [6,27,28]. 

### 2.2. Early Life Inflammation

Exposures that result in dysregulated immune responses during early life may have long-term consequences for health and disease. This time is a critical window of immunological development where critical changes are occurring in the developing immune system. Additionally, leukocytes take residence in adipose tissue during this time [29,30]. Chronic and cumulative inflammation is raised in obesity, and a risk factor for NCD, and early life infections, stress, and rapid weight gain can all contribute to cumulative inflammation (Figure 1B). Infants who are breastfed have overall lower inflammation. High sensitivity C-reactive protein (hsCRP), an inflammatory marker, has been used as a marker of cardiovascular disease risk in adults, and breastfed infants have 30% lower hsCRP than their formula-fed counterparts at 12 months of age [31,32]. In addition, a study of 26-year-old women identified that those who were breastfed had lower CRP than those who were never breastfed [33]. 

### 2.3. Early Life Metabolism

The establishment of metabolic physiology occurs early in life and lipid dysregulation is increasingly being recognised as a driver of obesity and cardiovascular disease risk [34,35]. Plasma lipidomics in adults has revealed that the dysregulation of lipid metabolism is associated with the initiation and progression of many NCD. The clinical blood lipid panel of children differs based on early life feeding practice, highlighting nutritional, and possibly metabolic, differences (Figure 1C). Breastfed infants initially have higher cholesterol, higher low-density lipoprotein, and lower high-density lipoprotein than formula-fed infants, which normalises by six months of age, and lower cholesterol in adulthood [36,37]. Moreover, a more complex analysis of the plasma lipidome of breastfed infants at three months of age identified lower total phosphatidylcholines (PC), lower short chain unsaturated fatty acid containing-PC, higher long chain polyunsaturated fatty acid containing- PC, higher cholesterol esters and differing sphingomyelin compared to infants who were not breastfed [9]. There is emerging evidence that breastfeeding significantly impacts the plasma lipidome through the first year of life, with some circulating lipid species, such as alkyldiacylglcyerols, present up to 17 times higher in those who were breastfed [12]. Although the exact effects of this on future disease risk remains unclear, prolonged breastfeeding duration is overall related to a healthier plasma lipidome in childhood and through teenage years. As such, breastfed infants have likely developed a beneficial metabolic and hormonal response to feeding which persists in later life [38]. 

## 3. The Emerging Field of Human Milk Lipidomics and Non-Communicable Disease Risk

**Figure 2:**
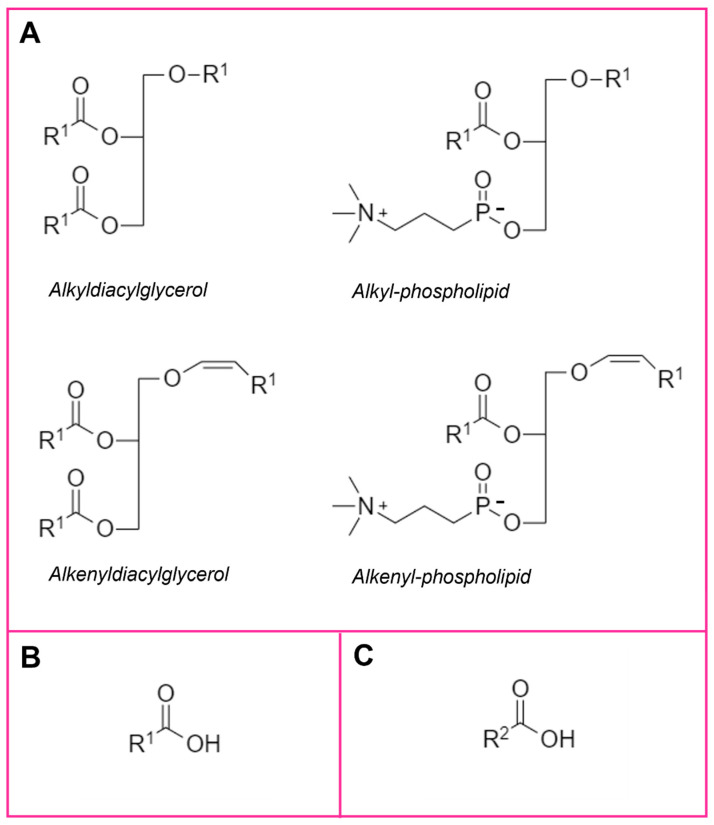
Chemical class structures for the bioactive lipids and lipid metabolites discussed in this review. (**A**) Ether lipids, note: alkyldiacylglcyerols may be saponified and measured as monoalkylglycerols. (**B**) Fatty acids. (**C**) Lipid metabolites, R^1^ carbon chain. R^2^ metabolised carbon chain.

Human milk is comprised of a multitude of nutritive and bioactive factors, including, but not limited to, lipids, proteins, carbohydrates, microbiome, immunoglobulins, and hormones. Lipids comprise approximately five percent of the total milk profile, the second most prevalent macronutrient after carbohydrates, yet provide over 50% of the energy to the infant [39]. The human milk lipidome is a complex mixture of many hundreds of lipid species across dozens of classes and subclasses, that together form human milk fat globules. These fat globules are comprised of a neutral lipid core, surrounded by a bilayer membrane containing cholesterol, phospholipids, and other lipids, as well as lipid metabolites, proteins, and peptides [40]. Figure 2 provides examples of the lipid species that will be discussed in more detail in this review. Human milk lipidomics, the identification and study of all individual lipid species that comprise the human milk lipidome, is a relatively recent field, having gained considerable traction over the last five years [17,41,42,43,44,45]. Various human milk lipids have been associated with infant growth parameters, but the underlying mechanisms or long-term effects of these on the infant are poorly understood.

## 4. Human Milk Ether Lipids

Ether lipids (Figure 2A) are a group of glycerol- or glycerophospho- lipids that contain an ether or vinyl ether linkage in the sn-1 position of the glycerol backbone, including alkylglycerols and plasmalogens. In humans, dietary alkylglycerols can be metabolised to plasmalogens, through a complex process involving alkylglycerol kinase, gastric lipase and pancreatic lipase. Plasmalogens are a highly bioactive ether lipid and are critical in human health due to their many biological functions. They are structural components of cells, potent antioxidants, and are critical to the differentiation of monocytes to macrophages, potentially modulating inflammatory processes. Plasmalogens also regulate cholesterol biosynthesis and efflux, and act as lipid mediators, providing a source of polyunsaturated fatty acids for subsequent production of eicosanoids and prostaglandins [46]. In adults, plasmalogens are altered in several disease states, reduced in obesity, type 2 diabetes and coronary artery disease [47,48,49]. 

Ether lipids are a relatively new area of interest in the context of early life development. Alkylglycerols are present in human milk but are negligible in most other food sources, and therefore do not exist in appreciable quantities in the adult diet [13,50,51]. When saponified, alkyldiacylglycerols lose their acyl chains at the sn-2 and sn-3 positions, leaving monoalkylglycerols, which have been measured in human milk as 16:0, 18:0 and 18:1 fatty alcohols, present in concentrations of approximately 3, 4 and 6 µmol/g protein, respectively. These data, albeit from a single human milk sample at two months postpartum, equate to approximately 27–36 µmol/L (O-16:0), 36–48 µmol/L (O-18:0) and 54–72 µmol/L (O-18:1) [13,52]. This study conducted complementary mouse experiments that showed alkylglycerols in mouse milk maintain and prolong the lifetime of beige adipocytes in pups, resulting in a higher beige adipose area, a higher mitochondrial count, a lower adipocyte size and a lower triacylglyceride content. This beige adipose maintenance occurred via the metabolism of alkylglycerols to platelet activating factor (PAF) in adipose tissue macrophages. This stimulates the PAF receptor, leading to interleukin-6 secretion and the activation of the STAT3 signalling pathway to promote differentiation. The upregulation of genes that reduce lipogenesis, promote lipid catabolism, form beige adipose tissue and promote thermoregulation within beige adipose tissue prevent the whitening of adipose [13]. In support of this, higher amounts of beige adipose tissue have been measured in children who were breastfed than those who were not, supplying evidence that human milk alkylglycerols may sustain beige adipose in humans [13]. Other forms of ether lipids, such as plasmalogens, are also found in human milk. Human milk alkenylphosphatidylcholine PC(P-18:0/18:0) was found in significantly higher amounts for preterm infants receiving human milk on a faster growth trajectory than for those on a slower growth trajectory [41]. Similarly, a positive association has been reported in term infants between plasmalogen intake (mean PC-P concentrations ranged from 17 to 485 nmol/L; mean PC-P intakes ranged from 14 to 363 nmol/day) in the first three months of life and infant weight at three and six months of age; however, it remains to be investigated if these are independent of total lipid intake [42]. 

The findings that alkylglycerols in human milk may promote a differentiation of infant beige adipose tissue, and thus a growth trajectory that may reduce obesity risk, is a compelling finding regarding the mediating role of human milk lipids in the link between breastfeeding and protection against obesity [13]. Given that ether lipids are highly bioactive in adults, and that ether lipid supplementation can change the lipidome of adults to improve health, the translational potential of an increased mechanistic understanding of ether lipids in human milk is promising [53]. Ether lipids may provide infant protection through other mechanisms; for example, previous in vitro studies indicate that alkylglycerols enhance *Lactobacillus* proliferation, so they may also influence infant health through the gut microbiome [54]. Ether lipids are currently not present in many infant formulae, or are present in significantly lower concentrations than in human milk [11]. Further unravelling of the links between human milk ether lipids and infant growth and inflammation will be essential to identifying if they provide similar, or additional protective roles as they do in adults.

## 5. Human Milk Fatty Acids

Fatty acids are carboxylic acids, the primary building blocks of all lipids (Figure 2B). Many fatty acids have links to human health, obesity and NCD risk, most commonly omega three (*n*-3) fatty acids, which are generally anti-inflammatory, and omega six (*n*-6) fatty acids, which are pro-inflammatory. The balance of these fatty acids is essential in adult health, where a lower plasma *n*-6:*n*-3 ratio is associated with a reduced risk of obesity, inflammation, and NCD [55]. 

Fatty acids are present in human milk as constituents of lipids and, to a lesser extent, as free fatty acids, and are the most researched of all the human milk lipids [18,44,45,56,57]. The human milk *n*-6:*n*-3 ratio appears important in infant growth; a lower ratio is beneficial and is associated with an increased lean body mass at age four and five [43]. Similarly, arachidonic acid (*n*-6) to eicosapentaenoic acid (*n*-3) and docosahexaenoic acid (DHA; *n*-3) ratios in human milk have been associated with infant fat mass and percentage body fat [44]. Human milk individual fatty acids and ratios appear to be important in adipose development, with a likely mechanism involving peroxisome proliferator-activate receptor gamma (PPAR-γ). PPAR-γ, and other PPAR isoforms, are transcription factors involved in the regulation of many biological processes, including energy homeostasis and lipid and glucose metabolism. PPAR- γ is present predominantly in adipose tissue and regulates adipocyte differentiation via a phosphorylation pathway regulating fatty acid storage and glucose metabolism [7]. PPAR-γ expression is increased by breastfeeding duration and expression is associated with infant growth rate [58,59]. The role of human milk fatty acids in this context is further supported by differences (especially in omega-6 and omega-3 lipid species) in human milk from women with healthy weight compared to that from women who are overweight, obese and diabetic, whose infants have a higher risk of obesity and later NCD [45].

Fatty acids may also contribute to infant NCD risk through their modulation of inflammation. PPAR-γ is also involved in the inflammatory response, with an anti-inflammatory action, possibly through both the interference of pro-inflammatory factors and the prevention of inflammatory gene transcription [7]. It is possible that fatty acids also prevent inflammation through protection against infection. Individual fatty acids including oleic, eicosenoic, linoleic, linolenic, palmitoleic and arachidonic acids also may have direct effects on microbial virulence. For example, they are capable of inactivating lipid-enveloped viruses, as well as neutralising pathogenic bacteria, and these are typically present in human milk in total relative abundances ranging from less than one percent (for arachidonic acid) to over 40% (for oleic acid) [39]. The anti-viral and anti-bacterial properties originate from both the free fatty acids and the monoglycerides that result from intragastric triacylglyceride hydrolysis. Oleic acid and oleic acid-containing monoglycerides are the primary antimicrobial products of infant milk digestion protecting against infection risk [14]. In a cohort of mothers and infants with and without cold-like symptoms, there were significant changes to human milk palmitic acid amounts, and similarly, palmitic acid-, lauric acid- and linoleic acid-containing triacylglycerides were delivered to the healthy infant in higher amounts than they were to unwell infants (defined as those with cold-like symptoms on the day of sample collection; approximately 4.2 vs. 3.5 g/day, 9 vs. 7.7 g/day, and 1.25 vs. 1 g/day, respectively) [18,19]. 

Many other human milk fatty acid associations and possible pathways with infant growth and inflammatory status have been suggested, although the data are much fewer than other fatty acid findings. For example, it has been proposed that reduced exposure to human milk short chain fatty acids in early life may program both allergy and overweight risk through the action of short chain fatty acids on regulatory T and dendritic cells, and involvement in fat oxidation and synthesis, in breastfed infants [7,17]. Women with allergic disease (whose infants have a higher risk of also developing allergic disease) have significantly lower concentrations of human milk acetate and butyrate than those of non-atopic mothers [17]. However, the extent to which this is prevalent is unclear, likely due to confounding factors, as another study found no link between human milk fatty acids and infant wheeze and asthma [56]. While short chain fatty acids are being delivered to the infant in human milk, believed to originate from maternal circulation, human milk oligosaccharides are also metabolised in the infant gut to produce short chain fatty acids, and is therefore another consideration in understanding the role they play in infant health [17]. Another possible way *n*-3 fatty acids may be involved in infant immunity is through their positive association with IgA and sCD14 in human milk [60].The fatty acid composition of different infant formulae varies significantly, and in comparison with human milk, typically depending on the lipid source used for formulation [10]. Fatty acids in the adult diet do have an important role in adult health, so it is reasonable that human milk fatty acids would play an important role in infant health, and this area will continue to produce interesting findings. 

## 6. Human Milk Lipid Metabolites

The lipokine 12,13-dihydroxyoctadecanoic acid (12,13-diHOME), is a metabolite of linoleic acid, produced in adipose tissue by the action of CYP2 and epoxide hydrolase enzymes (Figure 2C). It is released from adipose tissue in response to exercise and cold exposure [61]. 

In human milk, 12,13-diHOME has been reported at concentrations of 0.3–53.72 ng/mL. Following maternal exercise, at 1 month postpartum, the concentration of human milk 12,13-diHOME can increase approximately 1.4-fold, and higher 12,13-diHOME is associated with lower infant subcutaneous fat, and slower increase in BMI in the first six months of infancy. In the same study, other oxidised linoleic acid metabolites in human milk, 12,13-epoxyoctadecanoic acid and 9,10-dihydroxyoctadecanoic acid, were inversely associated with infant body fat at one month of age [16]. In mice, an injection of 12,13-diHOME increased fatty acid uptake into brown adipose tissue via fatty acid transporters in brown adipocytes, promoting thermogenesis and reducing BMI and insulin resistance [61]. A human cohort study showed it may be involved in the regulation of energy metabolism and of brown adipose activation, with higher 12,13-diHOME levels associated with lower plasma triacylglycerides and insulin [20]. It also appears to have a cardioprotective role, reducing the negative effects of a high-fat diet on mice, supported by human studies which demonstrated patients with heart disease had lower 12,13-diHOME than those without [22]. Through the promotion of ideal adiposity and growth, human milk 12,13-diHOME may also offer this protection from obesity and NCD risk. 

Further to this, increased gut bacteria-derived 12,13-diHOME (measured in infant faeces) is correlated with infant eczema, asthma and allergy. This suggests that human milk 12,13-diHOME could also have a role in immune regulation or function, but this is not yet clear in infant health [62]. 12,13-diHOME has previously been shown to play a role in the immune system and is elevated in the airways of adults who have asthma, both at the baseline and following stimulation with allergens, and in cases of acute respiratory distress syndrome [63]. This results from upregulation of pro-inflammatory LOX pathways and represents a basal level of inflammation present in these respiratory and allergic conditions [63]. 12,13-diHOME has been previously detected in bovine-milk based infant formula, in concentrations similar to the upper tertile of those found in human milk from healthy women but has not been further investigated in relation to infant growth [16]. The specific role of 12,13-diHOME remains unclear in infants but given its link to health in adults it is plausible that it offers critical protection in early life. 

Interestingly, another lipid metabolite, lysophosphatidylcholine (LPC) 14:0, has recently been presented as a link to infant obesity risk. Human milk fatty acids, including myristic acid (C14:0), influence infant serum LPC 14:0, which has previously been linked to rapid growth and obesity risk in childhood, and thus may also be a key lipid metabolite in future understandings of the impact of human milk on lipid metabolism [15,64]. 

## 7. Future Directions

The link between breastfeeding and obesity is well-established, but the mechanisms are not clear, which has hampered translation [5,21,23,65]. It is clear that human milk lipids and lipid metabolites play important roles in modulating the risk of obesity later in life through multiple mechanisms (Figure 1). This is especially relevant, given the rising importance of lipids in human health, and their therapeutic potential. Key focus areas for future research are shown in Box 1.

Box 1 **Future focus areas for understanding the role of human milk lipids and lipid metabolites in obesity, inflammation and non-communicable disease risk protection.**

Human milk lipids and lipid metabolites

❖ should be considered bioactive and included in early life studies
❖ need to be described comprehensively, in diverse populations
❖ need to be considered in combination with other biofluids, such as infant blood samples
❖ metabotypes or scores need to be developed and interrogated
❖ must be included within multidisciplinary research

To date, a comprehensive knowledge and understanding of the human milk lipidome is lacking, particularly in relation to infant outcomes and mechanisms. Limited studies have profiled some of the human milk lipidome, most commonly identifying around 300 lipid species, and up to around 700 (tentatively identified) lipid features over multiple analytical methods, across a limited number of Australian, Asian and European populations [11,19,42,57,66]. While these studies cover some geographical differences, specific details regarding maternal genetics, health and diet are mostly lacking. Future research should strive to collate more maternal data, including dietary information, and study diverse cohort populations to further the knowledge on the impact these have on the human milk lipidome and subsequent infant health. Further to this, sampling introduces major degrees of lipid variation, and thus comprehensive sampling is required to make use of such a lipidomics strategy, and sampling remains an important puzzle piece for interpreting and understanding the dynamics of the human milk lipidome. The variability and importance of sampling has previously been described; briefly, concentrations of single undefined human milk samples will differ greatly depending on time of day, time in lactation and if it was collected before, during or after a feed [67]. Furthermore, it appears that Holder pasteurization does not change the total lipid composition; however, there is no evidence to show whether individual lipids are altered by this, or other, processing methods. This is an essential consideration in the context of milk donations to ensure that these bioactive species are delivered to infants receiving donor milk [68]. Infants also consume different human milk volumes, which means that it is still quite difficult to standardise sampling, compare studies and link human milk lipids and lipid metabolites to infant outcomes [69]. With all of this in mind, studies are often limited by the number of human milk samples collected, and how detailed the collections are. For example, the evidence for alkylglycerols sustaining infant beige adipose tissue is interesting; however, the analysis was only carried out in a single milk sample from a single participant and thus a more thorough investigation is warranted to understand their importance and possible trends throughout lactation [13]. 

To better understand the role of human milk lipids and lipid metabolites, other biofluids, including infant blood samples, should be incorporated carefully into longitudinal studies. It is not clear exactly how human milk lipid intake may directly influence the infant plasma lipidome (circulating lipids) and additional sample types would help to define these metabolic links. Findings in blood are limited to very few studies which predominantly demonstrate the differences in the lipidome between breastfed and formula-fed infants [9,70]. It remains to be seen whether low *n*-6:*n*-3 ratios in human milk, for example, lead to the desired low *n*-6:*n*-3 blood ratio in the infant, which is known to be protective in adults. Although only briefly mentioned in this review, interactions between human milk lipids and gut microbiome, through faecal samples, will also be an important focus, given the importance of human milk oligosaccharides and the microbiome in early life growth and inflammation [54]. 

The integration of lipidomics in human health research has led to the emergence of metabotypes (defining population sub-groups by similarities in their metabolic profiles) or scores (assigning score values based on metabolic or phenotypic profiles) to interpret results, allowing an interrogation of the entire dataset, rather than a simple interrogation of individual species within a large dataset [35,71]. The incorporation and improvement of these will be required for human milk lipidomics, and an overall understanding of human milk components and their complex interactions. Distinct overall human milk lipid patterns have been identified between slow- and fast-growing infants, both term and preterm, with associations capable of predicting the growth group [71,72]. These analyses require an advanced bioinformatics approach and signpost the direction of future work to ensure that complex causal relationships can be defined between human milk lipids, infant health and NCD risk.

Finally, a more sophisticated multidisciplinary and holistic approach is required, incorporating multiple milk components, additional feeding data, clinical data and immune profiling to understand these complex relationships and to account for confounding factors. 

## 8. Conclusions

Human milk lipids and lipid metabolites are emerging as key candidates involved in the protection of the infant against obesity, inflammation and NCD. To understand their protective functions, human milk lipids and lipid metabolites need to be recognised as bioactive and new profiling strategies will be required to understand these lipids at an individual species level, and as a system of many different components in human cohorts. High quality future work in this field will ultimately contribute to our understanding of early life health and development, and to the prevention of obesity, inflammation, and NCD later in life. 

## Data Availability

Not applicable.

## References

[1] Wang Y., Wang J. (2020). Modelling and prediction of global non-communicable diseases. BMC Public Health.

[2] Moreno-Mendez E., Quintero-Fabian S., Fernandez-Mejia C., Lazo-de-la-Vega-Monroy M.-L. (2020). Early-life programming of adipose tissue. Nutr. Res. Rev..

[3] Geserick M., Vogel M., Gausche R., Lipek T., Spielau U., Keller E., Pfäffle R., Kiess W., Körner A. (2018). Acceleration of BMI in Early Childhood and Risk of Sustained Obesity. N. Engl. J. Med..

[4] Victora C.G., Bahl R., Barros A.J., França G.V., Horton S., Krasevec J., Murch S., Sankar M.J., Walker N., Rollins N.C. (2016). Breastfeeding in the 21st century: Epidemiology, mechanisms, and lifelong effect. Lancet.

[5] Yan J., Liu L., Zhu Y., Huang G., Wang P.P. (2014). The association between breastfeeding and childhood obesity: A meta-analysis. BMC Public Health.

[6] Azad M.B., Vehling L., Chan D., Klopp A., Nickel N.C., McGavock J.M., Becker A.B., Mandhane P.J., Turvey S.E., Moraes T.J. (2018). Infant Feeding and Weight Gain: Separating Breast Milk from Breastfeeding and Formula from Food. J. Pediatr..

[7] Stienstra R., Duval C., Müller M., Kersten S. (2007). PPARs, Obesity, and Inflammation. PPAR Res..

[8] Frank N.M., Lynch K.F., Uusitalo U., Yang J., Lönnrot M., Virtanen S.M., Hyöty H., Norris J.M., Rewers M., Bautista K. (2019). The relationship between breastfeeding and reported respiratory and gastrointestinal infection rates in young children. BMC Pediatr..

[9] Prentice P., Koulman A., Matthews L., Acerini C.L., Ong K.K., Dunger D.B. (2015). Lipidomic Analyses, Breast- and Formula-Feeding, and Growth in Infants. J. Pediatr..

[10] Zhang X., Liu L., Wang L., Pan Y., Hao X., Zhang G., Li X., Hussain M. (2021). Comparative Lipidomics Analysis of Human Milk and Infant Formulas Using UHPLC-Q-TOF-MS. J. Agric. Food Chem..

[11] Hewelt-Belka W., Garwolińska D., Młynarczyk M., Kot-Wasik A. (2020). Comparative Lipidomic Study of Human Milk from Different Lactation Stages and Milk Formulas. Nutrients.

[12] Burugupalli S., Smith A.A.T., Olshansky G., Huynh K., Giles C., Paul S., Nguyen A., Duong T., Mellett N., Cinel M. (2021). Ontogeny of plasma lipid metabolism in pregnancy and early childhood: A longitudinal population study. eLife.

[13] Yu H., Dilbaz S., Coßmann J., Hoang A.C., Diedrich V., Herwig A., Harauma A., Hoshi Y., Moriguchi T., Landgraf K. (2019). Breast milk alkylglycerols sustain beige adipocytes through adipose tissue macrophages. J. Clin. Investig..

[14] Isaacs C.E., Kashyap S., Heird W.C., Thormar H. (1990). Antiviral and antibacterial lipids in human milk and infant formula feeds. Arch. Dis. Child..

[15] Hellmuth C., Uhl O., Demmelmair H., Grunewald M., Auricchio R., Castillejo G., Korponay-Szabo I.R., Polanco I., Roca M., Vriezinga S.L. (2018). The impact of human breast milk components on the infant metabolism. PLoS ONE.

[16] Wolfs D., Lynes M.D., Tseng Y.H., Pierce S., Bussberg V., Darkwah A., Tolstikov V., Narain N.R., Rudolph M.C., Kiebish M.A. (2021). Brown Fat-Activating Lipokine 12,13-diHOME in Human Milk Is Associated with Infant Adiposity. J. Clin. Endocrinol. Metab..

[17] Stinson L.F., Gay M.C.L., Koleva P.T., Eggesbø M., Johnson C.C., Wegienka G., du Toit E., Shimojo N., Munblit D., Campbell D.E. (2020). Human Milk from Atopic Mothers Has Lower Levels of Short Chain Fatty Acids. Front. Immunol..

[18] Gardner A.S., Rahman I.A., Lai C.T., Hepworth A., Trengove N., Hartmann P.E., Geddes D.T. (2017). Changes in Fatty Acid Composition of Human Milk in Response to Cold-Like Symptoms in the Lactating Mother and Infant. Nutrients.

[19] George A.D., Gay M.C.L., Wlodek M.E., Trengove R.D., Murray K., Geddes D.T. (2020). Untargeted lipidomics using liquid chromatography-ion mobility-mass spectrometry reveals novel triacylglycerides in human milk. Sci. Rep..

[20] Vasan S.K., Noordam R., Gowri M.S., Neville M.J., Karpe F., Christodoulides C. (2019). The proposed systemic thermogenic metabolites succinate and 12,13-diHOME are inversely associated with adiposity and related metabolic traits: Evidence from a large human cross-sectional study. Diabetologia.

[21] Rito A.I., Buoncristiano M., Spinelli A., Salanave B., Kunešová M., Hejgaard T., García Solano M., Fijałkowska A., Sturua L., Hyska J. (2019). Association between Characteristics at Birth, Breastfeeding and Obesity in 22 Countries: The WHO European Childhood Obesity Surveillance Initiative—COSI 2015/2017. Obes. Facts.

[22] Pinckard K.M., Shettigar V.K., Wright K.R., Abay E., Baer L.A., Vidal P., Dewal R.S., Das D., Duarte-Sanmiguel S., Hernández-Saavedra D. (2021). A Novel Endocrine Role for the BAT-Released Lipokine 12,13-diHOME to Mediate Cardiac Function. Circulation.

[23] Horta B.L., Loret de Mola C., Victora C.G. (2015). Long-term consequences of breastfeeding on cholesterol, obesity, systolic blood pressure and type 2 diabetes: A systematic review and meta-analysis. Acta Paediatr..

[24] Rollins N.C., Bhandari N., Hajeebhoy N., Horton S., Lutter C.K., Martines J.C., Piwoz E.G., Richter L.M., Victora C.G. (2016). Why invest, and what it will take to improve breastfeeding practices?. Lancet.

[25] Mihrshahi S., Baur L.A. (2018). What exposures in early life are risk factors for childhood obesity?. J. Paediatr. Child Health.

[26] Dewey K.G., Peerson J.M., Brown K.H., Krebs N.F., Michaelsen K.F., Persson L.A., Salmenpera L., Whitehead R.G., Yeung D.L. (1995). Growth of breast-fed infants deviates from current reference data: A pooled analysis of US, Canadian, and European data sets. World Health Organization Working Group on Infant Growth. Pediatrics.

[27] Carberry A.E., Colditz P.B., Lingwood B.E. (2010). Body Composition from Birth to 4.5 Months in Infants Born to Non-Obese Women. Pediatric Res..

[28] Chomtho S., Wells J.C., Williams J.E., Davies P.S., Lucas A., Fewtrell M.S. (2008). Infant growth and later body composition: Evidence from the 4-component model. Am. J. Clin. Nutr..

[29] Prescott S.L. (2013). Early-life environmental determinants of allergic diseases and the wider pandemic of inflammatory noncommunicable diseases. J. Allergy Clin. Immunol..

[30] Olson J.S., Hayward M.D. (2017). Breastfeeding, overweight status, and inflammation. Soc. Sci. Res..

[31] McDade T.W., Metzger M.W., Chyu L., Duncan G.J., Garfield C., Adam E.K. (2014). Long-term effects of birth weight and breastfeeding duration on inflammation in early adulthood. Proc. R. Soc. B Biol. Sci..

[32] Ridker P.M. (2016). A Test in Context: High-Sensitivity C-Reactive Protein. J. Am. Coll. Cardiol..

[33] Williams M.J., Williams S.M., Poulton R. (2006). Breast feeding is related to C reactive protein concentration in adult women. J. Epidemiol. Community Health.

[34] Meikle P.J., Summers S.A. (2017). Sphingolipids and phospholipids in insulin resistance and related metabolic disorders. Nat. Rev. Endocrinol..

[35] Beyene H.B., Olshansky G., Giles C., Huynh K., Cinel M., Mellett N.A., Smith A.A.T., Shaw J.E., Magliano D.J., Meikle P.J. (2021). Lipidomic Signatures of Changes in Adiposity: A Large Prospective Study of 5849 Adults from the Australian Diabetes, Obesity and Lifestyle Study. Metabolites.

[36] Harit D., Faridi M.M., Aggarwal A., Sharma S.B. (2008). Lipid profile of term infants on exclusive breastfeeding and mixed feeding: A comparative study. Eur. J. Clin. Nutr..

[37] Kallio M.J.T., Salmenperä L., Siimes M.A., Perheentupa J., Miettinen T.A. (1992). Exclusive Breast-Feeding and Weaning: Effect on Serum Cholesterol and Lipoprotein Concentrations in Infants During the First Year of Life. Pediatrics.

[38] Hui L.L., Kwok M.K., Nelson E.A.S., Lee S.L., Leung G.M., Schooling C.M. (2019). Breastfeeding in Infancy and Lipid Profile in Adolescence. Pediatrics.

[39] Koletzko B. (2016). Human Milk Lipids. Ann. Nutr. Metab..

[40] Lopez C., Ménard O. (2011). Human milk fat globules: Polar lipid composition and in situ structural investigations revealing the heterogeneous distribution of proteins and the lateral segregation of sphingomyelin in the biological membrane. Colloids Surf. B Biointerfaces.

[41] Alexandre-Gouabau M.C., Moyon T., Cariou V., Antignac J.P., Qannari E.M., Croyal M., Soumah M., Guitton Y., David-Sochard A., Billard H. (2018). Breast Milk Lipidome Is Associated with Early Growth Trajectory in Preterm Infants. Nutrients.

[42] George A.D., Gay M.C.L., Selvalatchmanan J., Torta F., Bendt A.K., Wenk M.R., Murray K., Wlodek M.E., Geddes D.T. (2021). Healthy Breastfeeding Infants Consume Different Quantities of Milk Fat Globule Membrane Lipids. Nutrients.

[43] Meyer D.M., Brei C., Stecher L., Much D., Brunner S., Hauner H. (2019). Associations between long-chain PUFAs in maternal blood, cord blood, and breast milk and offspring body composition up to 5 years: Follow-up from the INFAT study. Eur. J. Clin. Nutr..

[44] Rudolph M.C., Young B.E., Lemas D.J., Palmer C.E., Hernandez T.L., Barbour L.A., Friedman J.E., Krebs N.F., MacLean P.S. (2017). Early infant adipose deposition is positively associated with the n-6 to n-3 fatty acid ratio in human milk independent of maternal BMI. Int. J. Obes..

[45] Mäkelä J., Linderborg K., Niinikoski H., Yang B., Lagström H. (2013). Breast milk fatty acid composition differs between overweight and normal weight women: The STEPS Study. Eur. J. Nutr..

[46] Paul S., Lancaster G.I., Meikle P.J. (2019). Plasmalogens: A potential therapeutic target for neurodegenerative and cardiometabolic disease. Prog. Lipid Res..

[47] Weir J.M., Wong G., Barlow C.K., Greeve M.A., Kowalczyk A., Almasy L., Comuzzie A.G., Mahaney M.C., Jowett J.B.M., Shaw J. (2013). Plasma lipid profiling in a large population-based cohort. J. Lipid Res..

[48] Meikle P.J., Wong G., Tsorotes D., Barlow C.K., Weir J.M., Christopher M.J., MacIntosh G.L., Goudey B., Stern L., Kowalczyk A. (2011). Plasma Lipidomic Analysis of Stable and Unstable Coronary Artery Disease. Arterioscler. Thromb. Vasc. Biol..

[49] Meikle P.J., Wong G., Barlow C.K., Weir J.M., Greeve M.A., MacIntosh G.L., Almasy L., Comuzzie A.G., Mahaney M.C., Kowalczyk A. (2013). Plasma Lipid Profiling Shows Similar Associations with Prediabetes and Type 2 Diabetes. PLoS ONE.

[50] Hallgren B., Niklasson A., Ställberg G., Thorin H. (1974). On the occurrence of 1-O-alkylglycerols and 1-O-(2-methoxyalkyl)glycerols in human colostrum, human milk, cow’s milk, sheep’s milk, human red bone marrow, red cells, blood plasma and a uterine carcinoma. Acta Chem. Scand. Ser. B Org. Chem. Biochem..

[51] Garcia C., Lutz N.W., Confort-Gouny S., Cozzone P.J., Armand M., Bernard M. (2012). Phospholipid fingerprints of milk from different mammalians determined by 31P NMR: Towards specific interest in human health. Food Chem..

[52] Ballard O., Morrow A.L. (2013). Human milk composition: Nutrients and bioactive factors. Pediatr. Clin. N. Am..

[53] Paul S., Smith A.A.T., Culham K., Gunawan K.A., Weir J.M., Cinel M.A., Jayawardana K.S., Mellett N.A., Lee M.K., Murphy A.J. (2021). Shark liver oil supplementation enriches endogenous plasmalogens and reduces markers of dyslipidemia and inflammation. J. Lipid Res..

[54] Chorostowska-Wynimko J., Krotkiewski M., Radomska-Leśniewska D., Sokolnicka I., Skopińska-Rózewska E. (2001). The synergistic effect of lactic acid bacteria and alkylglycerols on humoral immunity in mice. Int. J. Tissue React..

[55] Simopoulos A.P. (2011). Importance of the Omega-6/Omega-3 Balance in Health and Disease: Evolutionary Aspects of Diet. World Rev. Nutr. Diet..

[56] Logan C.A., Brandt S., Wabitsch M., Brenner H., Wiens F., Stahl B., Marosvölgyi T., Decsi T., Rothenbacher D., Genuneit J. (2017). New approach shows no association between maternal milk fatty acid composition and childhood wheeze or asthma. Allergy.

[57] George A.D., Gay M.C.L., Wlodek M.E., Murray K., Geddes D.T. (2021). The Fatty Acid Species and Quantity Consumed by the Breastfed Infant Are Important for Growth and Development. Nutrients.

[58] Mook-Kanamori D.O., Steegers E.A.P., Uitterlinden A.G., Moll H.A., van Duijn C.M., Hofman A., Jaddoe V.W.V. (2009). Breast-Feeding Modifies the Association of PPARγ2 Polymorphism Pro12Ala With Growth in Early Life: The Generation R Study. Diabetes.

[59] Rees W.D., McNeil C.J., Maloney C.A. (2008). The Roles of PPARs in the Fetal Origins of Metabolic Health and Disease. PPAR Res..

[60] Dunstan J.A., Roper J., Mitoulas L., Hartmann P.E., Simmer K., Prescott S.L. (2004). The effect of supplementation with fish oil during pregnancy on breast milk immunoglobulin A, soluble CD14, cytokine levels and fatty acid composition. Clin. Exp. Allergy.

[61] Lynes M.D., Leiria L.O., Lundh M., Bartelt A., Shamsi F., Huang T.L., Takahashi H., Hirshman M.F., Schlein C., Lee A. (2017). The cold-induced lipokine 12,13-diHOME promotes fatty acid transport into brown adipose tissue. Nat. Med..

[62] Levan S.R., Stamnes K.A., Lin D.L., Panzer A.R., Fukui E., McCauley K., Fujimura K.E., McKean M., Ownby D.R., Zoratti E.M. (2019). Elevated faecal 12,13-diHOME concentration in neonates at high risk for asthma is produced by gut bacteria and impedes immune tolerance. Nat. Microbiol..

[63] Lundström S.L., Yang J., Källberg H.J., Thunberg S., Gafvelin G., Haeggström J.Z., Grönneberg R., Grunewald J., van Hage M., Hammock B.D. (2012). Allergic Asthmatics Show Divergent Lipid Mediator Profiles from Healthy Controls Both at Baseline and following Birch Pollen Provocation. PLoS ONE.

[64] Rzehak P., Hellmuth C., Uhl O., Kirchberg F.F., Peissner W., Harder U., Grote V., Weber M., Xhonneux A., Langhendries J.P. (2014). Rapid Growth and Childhood Obesity Are Strongly Associated with LysoPC(14:0). Ann. Nutr. Metab..

[65] Dewey K.G., Güngör D., Donovan S.M., Madan E.M., Venkatramanan S., Davis T.A., Kleinman R.E., Taveras E.M., Bailey R.L., Novotny R. (2021). Breastfeeding and risk of overweight in childhood and beyond: A systematic review with emphasis on sibling-pair and intervention studies. Am. J. Clin. Nutr..

[66] Selvalatchmanan J., Rukmini A.V., Ji S., Triebl A., Gao L., Bendt A.K., Wenk M.R., Gooley J.J., Torta F. (2021). Variability of Lipids in Human Milk. Metabolites.

[67] George A.D., Gay M.C.L., Murray K., Muhlhausler B.S., Wlodek M.E., Geddes D.T. (2020). Human Milk Sampling Protocols Affect Estimation of Infant Lipid Intake. J. Nutr..

[68] Lamb R.L., Haszard J.J., Little H.M.J., Franks A.F., Meeks M.G. (2021). Macronutrient Composition of Donated Human Milk in a New Zealand Population. J. Hum. Lact..

[69] George A.D., Gay M.C.L., Wlodek M.E., Geddes D.T. (2021). The importance of infants’ lipid intake in human milk research. Nutr. Rev..

[70] Furse S., Billing G., Snowden S.G., Smith J., Goldberg G., Koulman A. (2019). Relationship between the lipid composition of maternal plasma and infant plasma through breast milk. Metab. Off. J. Metab. Soc..

[71] Alexandre-Gouabau M.C., Moyon T., David-Sochard A., Fenaille F., Cholet S., Royer A.L., Guitton Y., Billard H., Darmaun D., Rozé J.C. (2019). Comprehensive Preterm Breast Milk Metabotype Associated with Optimal Infant Early Growth Pattern. Nutrients.

[72] Jiang S., Pan J., Li Y., Ju M., Zhang W., Lu J., Lv J., Li K. (2021). Comprehensive Human Milk Patterns Are Related to Infant Growth and Allergy in the CHMP Study. Mol. Nutr. Food Res..

